# Modifying Hofstee standard setting for assessments that vary in difficulty, and to determine boundaries for different levels of achievement

**DOI:** 10.1186/s12909-016-0555-y

**Published:** 2016-01-28

**Authors:** Steven A. Burr, John Whittle, Lucy C. Fairclough, Lee Coombes, Ian Todd

**Affiliations:** 1 Collaboration for the Advancement of Medical Education Research and Assessment (CAMERA), Peninsula Schools of Medicine and Dentistry, Plymouth University, Devon, PL4 8AA UK; 2 School of Medicine, University of Nottingham, Queen’s Medical Centre, Nottingham, NG7 2UH UK; 3 School of Life Sciences, University of Nottingham, Queen’s Medical Centre, Nottingham, NG7 2UH UK; 4 Current address: Institute of Medical Education, School of Medicine, University of Cardiff, Cardiff, CF14 4YS UK

**Keywords:** Assessment, Hofstee, Standard setting, Satisfactory, Excellent, Grade, Ebel

## Abstract

**Background:**

Fixed mark grade boundaries for non-linear assessment scales fail to account for variations in assessment difficulty. Where assessment difficulty varies more than ability of successive cohorts or the quality of the teaching, anchoring grade boundaries to median cohort performance should provide an effective method for setting standards.

**Methods:**

This study investigated the use of a modified Hofstee (MH) method for setting unsatisfactory/satisfactory and satisfactory/excellent grade boundaries for multiple choice question-style assessments, adjusted using the cohort median to obviate the effect of subjective judgements and provision of grade quotas.

**Results:**

Outcomes for the MH method were compared with formula scoring/correction for guessing (FS/CFG) for 11 assessments, indicating that there were no significant differences between MH and FS/CFG in either the effective unsatisfactory/satisfactory grade boundary or the proportion of unsatisfactory graded candidates (p > 0.05). However the boundary for excellent performance was significantly higher for MH (*p* < 0.01), and the proportion of candidates returned as excellent was significantly lower (*p* < 0.01). MH also generated performance profiles and pass marks that were not significantly different from those given by the Ebel method of criterion-referenced standard setting.

**Conclusions:**

This supports MH as an objective model for calculating variable grade boundaries, adjusted for test difficulty. Furthermore, it easily creates boundaries for unsatisfactory/satisfactory and satisfactory/excellent performance that are protected against grade inflation. It could be implemented as a stand-alone method of standard setting, or as part of the post-examination analysis of results for assessments for which pre-examination criterion-referenced standard setting is employed.

## Background

Many university assessment systems have established pre-existing passing scores for determining degree classifications after application of an appropriate correction factor to account for guessing [1]. However, it is clear that variations in test difficulty have a marked effect on pass/fail rates for different cohorts [2], and thus predetermined fixed standards are increasingly difficult to justify and defend.

There is no single gold standard for setting grade boundaries for multiple choice question (MCQ)-style assessments, and criterion-based approaches (such as Angoff or Ebel) rely on panels of judges reviewing each question item [3]. However these criterion-based methods are resource intensive and susceptible to a high degree of inter-reviewer variability [4]. The alternative, norm-referenced, approaches involve failing a fixed proportion of each cohort and thus appear unfair. However norm-referencing is only unfair if there is significant variation in performance between cohorts, which is unlikely to be a significant factor [1]. It therefore seems reasonable to consider a method that sits somewhere between these two approaches.

The Hofstee method [5] can be described as a compromise between criterion-referenced and norm-referenced methods of standard setting, and is used in the UK to set standards on undergraduate exams [6]. While it is often not a first choice for many practitioners [7], it is still considered to be a common method and is reported in standard setting guides alongside more familiar methods such as Angoff and Ebel [8]. While there is evidence that the Hofstee method produces appropriate, stable and reliable passing scores [9, 10], concerns remain about its fairness and credibility [11].

**Fig. 1:**
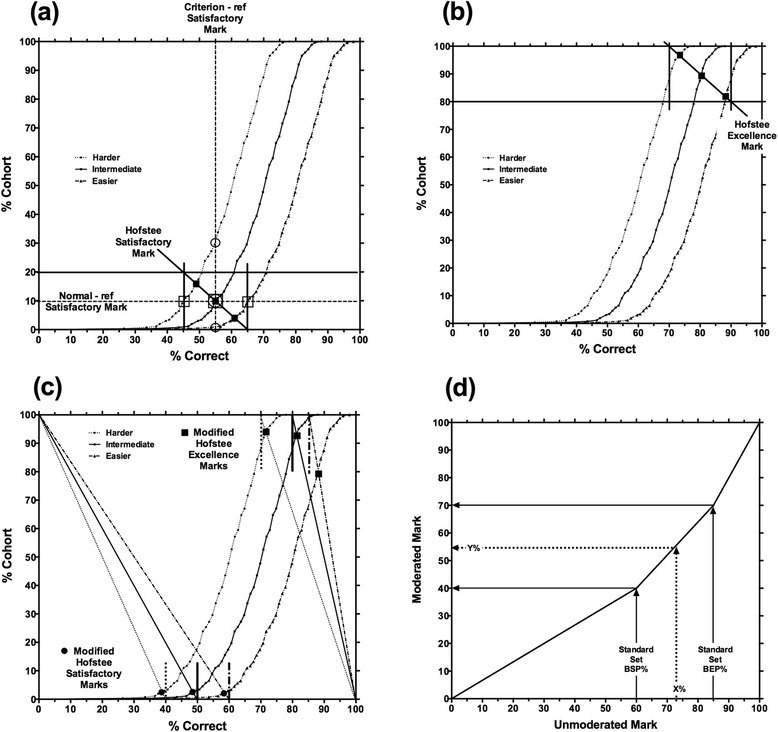
Model for Hofstee standard setting. **a** Performance curves for three assessments (harder, intermediate and easier); open circles indicate the percent of the cohort who fail each assessment with a criterion reference pass mark of 55 %; open squares indicate the pass marks for a norm-referenced failure rate of 10 % of the cohort; solid squares indicate the pass marks and percent of the cohort who fail by application of the Hofstee method. **b** Application of Hofstee criteria to determine a BEP (indicated by the solid squares) – see text for details. **c** Application of modified Hofstee criteria to determine BSP (solid circles) and BEP (solid squares) – see text for details. **d** Graphical presentation of ‘cranking’ the standard set marks from an assessment to moderated marks on the University scale where the pass mark of 40 % equates to the BSP% and the 70 % distinction/first-class mark equates to BEP%. An individual student’s standard set mark is mapped to the new moderated mark through linear interpolation on the gradient of the relevant line (e.g. X% mapped to Y%)

Figure 1a shows how passing scores are determined in the Hofstee method, and represents the results of three assessments of different levels of difficulty on a Hofstee plot. If a fixed passing score (criterion-referencing) was applied (represented by the vertical dashed line) then a very large difference is observed across the three assessments in the proportion of candidates who fail (indicated by the three open circles). If, on the other hand, a fixed proportion of the cohort were failed (norm-referencing, represented by the horizontal dashed line) then there is a large difference in the pass marks across the three assessments (indicated by the three open squares).

The application of the Hofstee method is represented by the bold solid lines in Fig. 1a. The vertical lines represent the maximum and minimum satisfactory boundary (i.e. the highest and lowest scores required to pass). The horizontal line represents the maximum percentage fails (i.e. the highest acceptable proportion of candidates failing (set at 20 % in this example); note that the minimum failure rate is set at 0 % as all of the candidates could pass). The effective passing score, or Boundary for Satisfactory Performance (BSP), is set to the point at which the diagonal line intersects with the curve for each assessment (indicated by the solid squares). For the intermediate assessment, the effective BSP is the same by all three methods. For the harder and easier assessments, the change in BSP (and consequent change in proportion of candidates who fail) by applying Hofstee is less than given by either the criterion or norm-referenced methods alone. Nevertheless, there remain perceived inequities that could be accounted for by modifying the Hofstee method to: control for differences in test difficulty; remove the percentage fail quotas; and, include additional grade boundaries.

This study aims to develop a modified form of the Hofstee method for MCQ-style assessments that does not require time-consuming and relatively subjective assessments of the difficulty of individual questions as in the Anghoff and Ebel methods, and which could be used to determine the boundary between satisfactory and excellent performance to produce a Boundary for Excellent Performance (BEP) as well as a BSP. Comparison of candidate outcomes with an established formula scoring or ‘correction for guessing’ (FS/CFG) method, which had been used for a number of years prior to the introduction of standard setting, should provide evidence to examine whether the modified Hofstee (MH) approach performs more appropriately. FS/CFG is not a standard setting method – it is simply a mechanism to moderate for guessing of correct responses; therefore, comparison is also made to the criterion-referenced Ebel standard setting method for determination of the BSP to establish whether the MH method performs similarly to this well-established standard setting method.

## Methods

### Applying the Hofstee method to determine a Boundary for Excellent Performance (BEP)

We propose that the criteria of the Hofstee method for determining a BSP (Background section, Fig. 1a) can be ‘inverted’ for the determination of a BEP (i.e. ‘first class’ or ‘distinction’). This is illustrated in Fig. 1b, employing the same three performance curves as in Fig. 1a. The vertical solid lines represent the maximum and minimum boundary for excellence marks (i.e. the highest and lowest scores required to be judged ‘first class’). The percentage above the horizontal solid line represents the maximum acceptable proportion of candidates judged to be ‘first class’ (set at 100–80 = 20 % in this example); the minimum proportion is 0 % as it is feasible that none of the candidates demonstrate ‘excellence’). The diagonal line then gives the effective Boundary for Excellent Performance (BEP) where it intersects with the curve for each assessment.

### Determining boundaries based on the median performance of a cohort

The essence of the classical Hofstee method is that judges decide on the minimum and maximum failure rate and acceptable pass mark. The pass mark range is based on the perceived difficulty of the assessment, with harder exams typically setting lower BSPs, though the exact range used is a subjective decision. However, if we assume that the students who take an assessment are sufficiently representative of the whole population of possible students and that the quality of the teaching is stable, then their performance can be used as a measure of the assessment difficulty. We verified this assumption by analysing response data for 31 multiple choice and extended matching questions (incorporating a total of 73 correct items and many more distractors) that were attempted by between three and six different cohorts of first year medical students in summative exams over a seven-year period (with between 237 and 263 students in each cohort). The percentage of students in the different cohorts that chose the correct items had a mean coefficient of variation of only 3.8 % (standard deviation ±3.0 %). This shows that correct response rates are stable across multiple cohorts of students over multiple years. We therefore propose that the BSP is adjusted based on the performance of the cohort as a whole, as judged by the median percentage mark (the median is used because cohort performances are frequently skewed with a tail caused by a small number of disproportionately low-scoring fails). Similarly, a BEP can be determined, as described in the previous paragraph, with its position set relative to the median performance of the cohort as a measure of assessment difficulty rather than setting an arbitrary boundary for excellence.

### Boundaries for modified Hofstee

We undertook initial modelling on historical assessment data in order to determine acceptable boundaries in relation to both the BSP and the BEP. The assessments were composed of objective multiple choice questions (MCQ) – this term is used here generically to include single best answer, multiple choice, extended matching questions, etc. These assessments were delivered in the open-source Rogo e-Assessment Management System (http://rogo-oss.nottingham.ac.uk/); they represented summative assessments from a range of biomedical science disciplines delivered in semesters 1–4 of a medical course.

We decided that the maximum and minimum proportion of the cohort who can either fail or achieve excellence should be set at 100 and 0 %, respectively, since it could be deemed unfair to set a limit. Thus, all students in a cohort would be deemed ‘unsatisfactory’ if none of them scored any marks; equally, all students in a cohort would be deemed ‘excellent’ if they all scored 100 %.

Several years’ experience of applying FS/CFG to MCQ assessments for a range of modules had shown that failure rates up to 10 % (but usually ≤5 %) on individual modules were acceptable in terms of level of difficulty and identifying students whose level of knowledge and understanding were unsatisfactory when applying a pass mark of 40 %. We therefore deemed that the MH protocol should be calibrated to generate a similar failure rate. We found that this was achieved by setting the maximum unsatisfactory mark at 20 percentage marks below the median percentage mark for the assessment, or setting it at 60 %, whichever is lower. Similarly, for an MSc module with a pass mark of 50 %, an acceptable failure rate (compared to FS/CFG) was achieved by setting the maximum unsatisfactory mark at 10 percentage marks below the median, or at 70 %, whichever was lower. In all cases, the minimum satisfactory mark is set at 0 %; the reason for this is that we wanted to avoid any application of FS/CFG as this automatically assumes an element of guessing, which may not always be the case even for candidates whose score is less than the FS/CFG mark. Setting the lower limit of the pass mark at zero then allows for the theoretical possibility that all the questions in an assessment were so difficult that any marks achieved should merit a pass, and that the number of distractors is so large that the likelihood of guessing a correct answer is insignificant (e.g. < 1/20).

We judged that, for most assessments, the proportion of candidates deemed to show excellent performance should be between 5 and 30 % of the cohort; we found that this was achieved when the minimum mark for excellence is set at 10 percentage marks above the median percentage mark for the assessment, or is set at 85 %, whichever is lower. The maximum mark for excellence is set at 100 %; this allows for the theoretical possibility that all the questions are sufficiently easy that demonstrating excellence requires all responses to be correct.

It should be noted that it is not appropriate to use a formula to determine proportions of performances that are deemed to be either unsatisfactory or excellent as this would tie the method to norm-referencing with fixed quotas in particular categories of performance; the MH method has been deliberately designed to avoid generating fixed quotas of unsatisfactory or excellent candidates. For example, it is possible that no student performances would be deemed unsatisfactory or excellent if the spread of marks around the median is very low – e.g. if the median mark was 75 % and the whole distribution of marks was between 55 and 85 %.

The ‘whichever is lower’ clause for the determination of both BSP and BEP is in the candidates’ favour. In addition, all percentage marks were calculated to two decimal places to plot the Hofstee cumulative frequency curves to avoid inaccuracies which might result from the premature rounding of marks. The effective boundaries (cut scores) determined for satisfactory and excellent performance (BSP and BEP, respectively) were then rounded to the whole percentage mark below the cut score to two decimal places (e.g. 56.87 would be rounded to 56) in order also to be in the candidates’ favour.

The application of the above principles for setting the boundaries (for assessments with a University pass mark of 40 %) is illustrated in Fig. 1c for the same sets of results shown in Fig. 1a and b. The median scores in the harder, intermediate and easier assessments are 60, 70 and 80 %, respectively; this generates upper limits for the BSP of 40, 50 and 60 %, respectively (median% minus 20 %). Diagonal lines are then drawn from these boundaries to the point where 100 % of the cohort would achieve 0 %, and the intersection of these diagonals with the performance curves gives the BSP. The three performance curves generate lower limits for the BEP of 70, 80 and 85 %; this represents the median% plus 10 %, except for the ‘easier’ assessment, where this would be greater than 85 %. Diagonal lines are then drawn to the point where 0 % of the cohort achieves 100 %, and the intersections with the performance curves give the BEP.

### Data analysis

Modified Hofstee (MH) standard setting was applied to the results of 8 independent summative MCQ assessments (Modules 1–8) all sat in the same academic session by year 1 or year 2 undergraduate (UG) medical student cohorts (258–266 students), and a further 2 optional modules (Modules 9–10) each sat by a different subset of the same year 2 UG cohort (25–29 students); all of these assessments had a University-scale UG BSP of 40 % and a BEP of 70 %; they represented summative assessments collectively comprising all biomedical science disciplines delivered in semesters 1–4 of a UG medical course. MH was also applied to the results of an MSc level 3 post-graduate (PG) module in basic immunology with a University-scale PG BSP of 50 % and a BEP of 70 % (Module 11); this assessment was taken by three separate cohorts (37–50 students). The study was approved by the Ethics Committee of The School of Life Sciences, Faculty of Medicine and Health Sciences, University of Nottingham, UK (Ethics Reference Number B181114IT). Participant consent was deemed not to be necessary for this anonymised assessment data and was exempted from the ethical approval process. Permission to use the assessment data (to which access is restricted) was granted by the Associate Dean for Medical Education, Faculty of Medicine and Health Sciences, University of Nottingham, on behalf of the University. The summative assessment results returned by MH were compared with the assessment results that would have been returned if FS/CFG was applied. Summary statistics were expressed as medians with interquartile ranges, and significance determined by Wilcoxon matched-pairs tests.

The random mark used to determine the FS/CFG was calculated for each question using the formula R = N^2^/T, where R is the random mark, N is the number of correct options, and T is the total number of options (where each correct option is worth one mark). For example, for a single best answer question with four options: R = 1^2^/4 = 0.25; for a multiple response question with two correct options from a choice of five: R = 2^2^/5 = 0.8. The overall random mark for an assessment was then calculated as the sum of the random marks for all the questions in the assessment. The FS/CFG-adjusted mark for each candidate was then calculated using the formula:

$$ \mathrm{F}\mathrm{S}/\mathrm{C}\mathrm{F}\mathrm{G}-\mathrm{adjusted}\ \mathrm{mark}=\left[\mathrm{marka}\ \mathrm{chieved}\ \hbox{--}\ \mathrm{random}\ \mathrm{mark}\right]\div \left[\mathrm{total}\ \mathrm{available}\ \mathrm{mark}\mathrm{s}\ \hbox{--}\ \mathrm{random}\ \mathrm{mark}\right]. $$

### Worked example

Table 1 shows a worked example of marks processing using the MH protocol for the module 10 assessment (taken by 29 students):

**Table 1:** Worked example of marks processing using the modified Hofstee protocol for the module 10 assessment

1 Raw mark (%)	2 MH Mark (%) after conversion to university boundaries (40 % BSP & 70 % BEP)	3 Mark with FS/CFG applied (%)
50	**38**	**31**
50	**38**	**31**
60	47	45
60	47	45
64	50	50
64	50	50
66	52	53
68	53	56
68	53	56
70	55	59
70	55	59
76	60	67
76	60	67
78	62	***70***
78	62	***70***
82	65	***75***
84	67	***78***
86	68	***81***
86	68	***81***
86	68	***81***
88	***70***	***83***
88	***70***	***83***
88	***70***	***83***
90	***75***	***86***
90	***75***	***86***
92	***80***	***89***
94	***85***	***92***
94	***85***	***92***
98	***95***	***97***

In columns 2 and 3, the marks below the BSP are shown in bold; the marks on or above the BEP are shown in bold italics

1. The marks expressed as percentages are shown in Table 1, column 1 to two decimal places (in this instance all the % marks are integers).
2. The median mark for the cohort is 78 %; applying the protocol described above, this sets the upper limit for the Boundary for Satisfactory Performance (BSP) at 58 % (20 % below the median is <60 %), and sets the lower limit for the Boundary for Excellent Performance (BEP) at 85 % (10 % above the median is >85 %).
3. The cumulative frequency curve of assessment marks (%) for percentage of the cohort (Y) against percentage correct score (X) is plotted (e.g. using the Survival Curve option in Graphpad Prism version 5.0d). This ‘performance’ curve of the cohort (% correct versus % of cohort achieving that mark or lower) is subjected to the MH protocol to determine the actual BSP and BEP (shown in Fig. 2b).

**Fig. 2:**
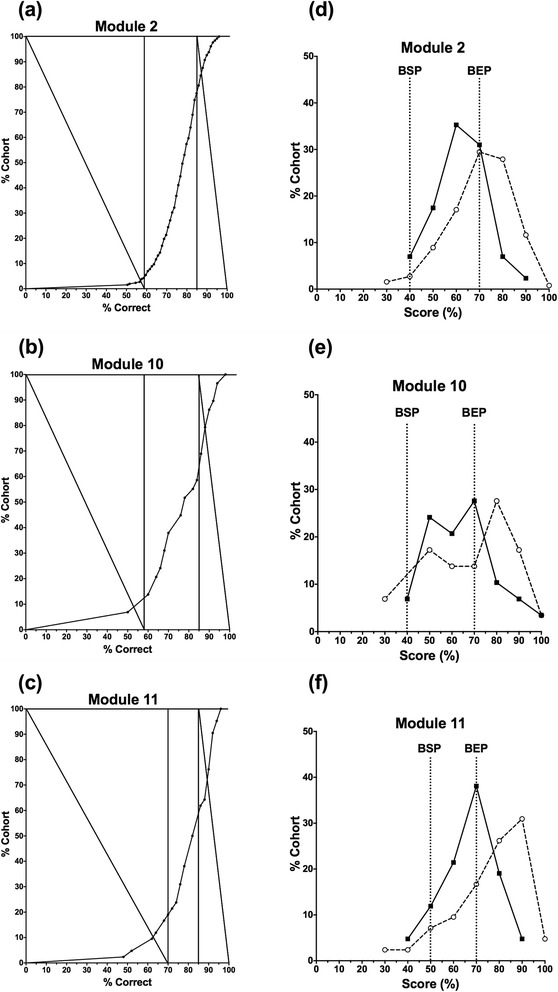
**a-c** Examples of applying the modified Hofstee protocol to the cumulative frequency curves of student cohort performance in MCQ-style assessments: **a** module 2; **b** module 10; **c** module 11. Modules 2 and 10 have a University pass mark of 40 % whereas module 11 has a University pass mark of 50 %; all three modules have a University first class/distinction mark of 70 %. **d-f** The frequency distributions of student performance in the same three assessments comparing the outcomes given by FS/CFG (dashed curves) and the MH protocol (solid curves) following moderation (‘cranking’) to the University scales – 40 %/70 % for modules 2 (**d**) and 10 (**e**), and 50 %/70 % for module 11 (**f**)
4. Where the diagonal lines cross the performance curve give: BSP = 52 % (rounded down from 52.83 % actual); BEP = 88 % (rounded down from 88.02 % actual) (Fig. 2b).
5. The candidates’ percentage marks (X%) are converted using an equation in Microsoft Excel to normalise BSP (52 %) to a university boundary of 40 % (minimum pass mark), and BEP (88 %) to a university boundary of 70 % (minimum first class or distinction mark) to derive their mark (Y%) as in Fig. 1d. This gives the final standard set percentage marks shown in Table 1, column 2.
6. For comparison, the equivalent percentage marks given using FS/CFG are shown in Table 1, column 3. In columns 2 and 3, the marks below the BSP are shown in bold; the marks on or above the BEP are shown in bold italics.

## Results

### Determining BSP and BEP using MH analysis

Figure 2(a-c) shows three examples of the cumulative frequency curves, each used to derive the Boundary for Satisfactory Performance (BSP) and Boundary for Excellent Performance (BEP) for a different individual module. Modules 2 and 10 have a University-scale UG BSP of 40 %, and module 11 has a University-scale PG BSP of 50 %; all have University-scale BEPs of 70 %.

### Comparison of MH and FS/CFG for determining satisfactory performance

**Fig. 3:**
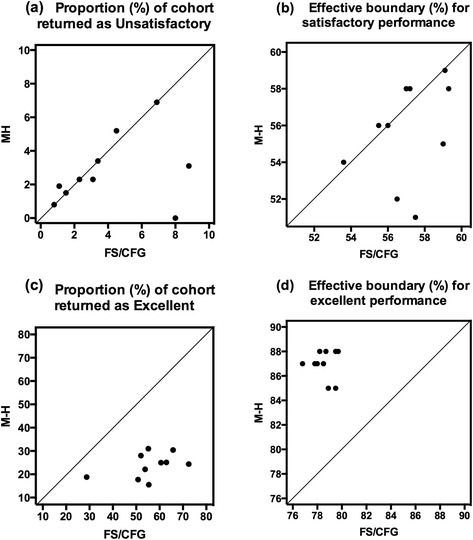
Comparison of the outcomes for applying MH or FS/CFG to the assessments of ten different UG modules: **a** the percentage of candidates showing unsatisfactory performance; **b** the percentage marks defining the effective boundary for satisfactory performance; **c** the percentage of candidates showing excellent performance; **d** the percentage marks defining the effective boundary for excellent performance

Figure 3a shows a comparison of MH with FS/CFG for determining satisfactory performance in the ten UG module assessments. There is no significant difference (p > 0.05) between the proportion of candidates returned as unsatisfactory: FS/CFG median = 3.25 % of candidates and MH median = 2.3 % of candidates. Thus, MH returns a proportion of candidates deemed to show unsatisfactory performance (fail) similar to that given by FS/CFG, when applying a maximum MH BSP of 60 %. It should be noted, however, that the interquartile range for the percentage of failing candidates is much lower using MH (3.850 – 1.325 = 2.525 %) than using FS/CFG (7.175 – 1.4 = 5.775 %). Given that all ten assessments were taken in the same academic session by first or second year medical students (or a subset thereof), this is consistent with the standard setting properties of MH taking account of exam difficulty, which FS/CFG does not.

The reason for the similar fail rates given by MH and FS/CFG is shown in Fig. 3b, which compares the BSP determined by MH with the ‘effective’ BSP using FS/CFG. The latter is the percentage of the total marks in the assessment that a candidate must achieve in order to be awarded a mark of 40 % after subtraction of the random mark from both the actual mark and the total marks; this is derived from the formula 0.4 = (effective BSP – random mark) / (total marks – random mark). There is no significant difference (p > 0.05) between the effective BSPs: FS/CFG median = 57.1 % (interquartile range = 55.88–59.03) and MH median = 56.0 % (interquartile range = 53.5–58.0).

### Comparison of MH and FS/CFG for determining excellent performance

Figure 3c shows a comparison of MH with FS/CFG for determining excellent performance in the ten UG module assessments. There is a significant difference (*p* < 0.01) between the proportion of candidates returned as demonstrating excellent performance: FS/CFG median = 55.3 % of candidates (interquartile range = 51.7–63.63) and MH median = 24.7 % (interquartile range = 18.53–28.6). Thus, MH returns a significantly lower proportion of candidates deemed to show excellent performance (first class, distinction) when applying a maximum lower limit of BEP of 85 %.

The reason for the very different rates of performance deemed to be ‘excellent’ given by MH and FS/CFG is also shown in Figure 3d, which compares the BEP determined by MH with the ‘effective’ BEP using FS/CFG. The latter is the percentage of the total marks in the assessment that a candidate must achieve in order to be awarded a mark of 70 % after subtraction of the random mark from both the actual mark and the total marks; this is derived from the formula 0.7 = (effective BEP – random mark) / (total marks – random mark). There is a significant difference (*p* < 0.01) between the effective BEPs: FS/CFG median = 78.6 % (interquartile range = 77.95–79.5) and MH median = 87 % (interquartile range = 86.5–88.0).

### Comparison of the marks profile generated by MH and FS/CFG

Figure 2(d-f) shows the frequency distribution of marks generated by MH (after moderation to the University scale with UG 40 %/70 % boundaries and PG 50 %/70 % boundaries) and FS/CFG, again using modules 2, 10 and 11 as examples. In both cases, FS/CFG gives marks distributions heavily skewed to the right, with the majority of candidates being awarded scores close to, or greater than, the BEP. The application of MH shifts the marks distribution to the left, generating a more symmetrical ‘normalised’ distribution; in most cases, the majority of candidates are then awarded scores between the BSP and BEP. In addition, lower performing candidates (with marks close to, or below, the BSP) are awarded somewhat higher scores using MH than with FS/CFG. The more favourable marks generated by MH for lower performing candidates is because FS/CFG assumes an element of guessing, so that a candidate whose actual mark is the same as the random mark is awarded 0 % if FS/CFG is applied.

### Correlation between marks awarded using MH or Ebel standard setting

All UK medical schools are now required to employ standard setting methodologies in their assessments [6]. It is therefore important to assess the validity of the MH method in comparison to a widely used, criterion-referenced standard setting method, such as that proposed by Ebel [12]. In this method, judges rate each question according to difficulty (easy/medium/hard) and relevance (essential/important/nice-to-know); this generates nine categories of questions (easy/essential, medium/important, etc.) and, for each, a judgement is made on the percentage of questions in each category that a ‘borderline candidate’ on the cusp of failing would be expected to answer correctly. Multiplying the number of marks associated with each category by the corresponding’borderline percentage correct’ and then adding up the values for all nine categories, gives the pass mark (BSP).

**Fig. 4:**
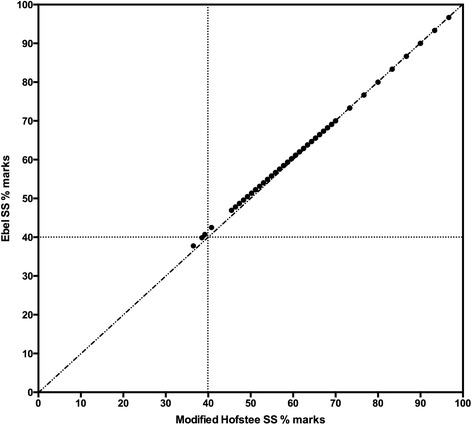
Correlation of the marks profile generated by applying Ebel or MH standard setting to a year 1 medical student assessment. Following standard setting by either method, the BSP was converted to a university-set pass mark of 40 % (indicate by the horizontal and vertical dotted lines on the graphs). The solid circles show the marks of candidates determined by both methods of standard setting

Figure 4 shows the marks generated for the assessment of a year 1 module using Ebel or MH standard setting to determine the BSP. A Pearson product–moment correlation coefficient between marks generated by Ebel and MH standard setting is highly significant (*r* = 0.9998, *n* = 262, *p* < .0001). Out of the 262 candidates, one would have failed by applying the Ebel BSP, and three would have failed by applying the MH BSP.

### Similar pass marks generated by MH and Ebel standard setting

As a further comparison between the MH and Ebel methods, data from interdisciplinary clinical MCQ assessments were analysed in terms of the BSP generated by Ebel standard setting panels of judges (the procedure implemented for the exams) and the MH method applied retrospectively to the assessment results. Each assessment was attempted by cohorts of >300 fifth year medical students, with two MCQ exams taken by each cohort; data over a five year period were analysed, i.e. ten assessments in total. For these high-stake, final year assessments, we determined that the upper limit of the MH BSP should be set at 15 percentage marks below the median percentage mark for the cohort. A paired samples *t*-test indicated there was no significant difference between the BSPs for each assessment generated by applying Ebel or MH standard setting (t(9) = 1.417, p = 0.1902); there was also no significant difference in the number of candidates who failed when applying the BSP generated by Ebel or MH standard setting (p = 0.7031 by Wilcoxon matched-pairs signed rank test).

## Discussion

We have described here a modification of the Hoftsee standard setting method that employs the median score of a cohort to determine cut scores rather than the time-consuming and relatively subjective decisions of a panel of judges. In addition, we have used this to determine different boundaries (e.g. unsatisfactory/satisfactory and satisfactory/excellent), although this principle could also be applied to the conventional Hofstee method or to other standard setting methods; indeed, we have previously used the Ebel method to standard set the satisfactory/excellent borderline (unpublished observations). The principles of the MH method could readily be applied to other cut-points and to other grading systems that are in use around the world, in addition to the standard UK scoring system exemplified here. Furthermore, although the MH method has been developed for MCQ-style assessments, the principles of the method could be adapted to other assessment formats.

When compared to outcomes based on a FS/CFG method, the MH method produced similar boundaries for satisfactory performance and similar proportions of unsatisfactory graded candidates. The boundary for excellent performance was significantly higher using the MH method, with a significantly lower proportion of candidates awarded excellent grades. Furthermore, the MH protocol generated marks profiles and BSPs very similar to those given by applying the Ebel method of criterion-reference standard setting.

A key feature of the MH protocol described here is that the upper limit of the BSP is set an absolute (rather than relative) distance below the median mark of the cohort. This has several advantages: it controls for variation in difficulty between assessments; bases the satisfactory/unsatisfactory boundary mark on the average performance of all the candidates (while accounting for a non-normal distribution); avoids the necessity to impose a quota (such as failing all those below the 95 % confidence interval). We have also demonstrated that the MH protocol allows appropriate BSPs to be established for assessments requiring demonstration of different levels of competence (e.g. a 40 or 50 % pass mark on the University scale). Furthermore, whereas others have reported that the conventional Hofstee method generates only small changes in BSP for relatively large changes in the boundaries for fail rates and pass marks [11], we have demonstrated that the MH method detailed in the present report generates much larger changes in actual BSP when the position of the upper limit of the BSP is varied relative to the median performance of the cohort [13]; this is intuitively consistent with the standard setting process.

In addition, our results demonstrate that, by establishing a standard set BEP an absolute distance above the cohort median, MH reduced the proportion of candidates being awarded excellent grades and consequently protected against ‘marks creep’. Thus, including control over the cut score for excellence mitigates against grade inflation and the danger of devaluing a qualification [14]. This appears to be particularly evident when using objectively marked assessments (e.g. multiple choice questions, extended matching questions, etc.), which involve identification of correct information, rather than ‘unprompted recall’ of correct information (e.g. short answer questions, essay questions, etc.). Furthermore, where conversion back to a range of marks correlating to university degree categories, determination of cut scores for both satisfactory and excellent performances increases fairness where the categories correlate to non-linear mark brackets (e.g. fail = 0–39 %, 3rd = 40–49, 2.2 = 50–59, 2.1 = 60–69, 1st = 70–100 %).

With regard to the time taken to apply the MH procedure, we have found that, from accessing the ‘raw’ marks to generating the final marks, standard set and converted to a university scale, takes about 15 min for each assessment. However this is considerably less labour intensive than criterion-only referenced methods (e.g. Ebel, Angoff), and MH has the further advantage that much of the process can be automated by computer. Indeed, programming of the MH method in the Rogo Assessment System means that the final, standard set results can be generated in 1–2 min. The pragmatic need for standard setting methodologies that are ‘affordable’ in terms of staff time and resources has also been a key factor in other standard setting protocols that have been reported recently [15]. Furthermore, in situations where pre-examination criterion-referenced standard setting methods, such as Ebel, are likely to be the method of choice (e.g. ‘high-stakes’ clinical exams), MH could be used as part of the post-examination analysis of results in the quality assurance of the whole standard setting process. If, for example, Ebel and MH generated significantly different pass marks and/or numbers of fails for the same assessment, further investigation could then be undertaken to determine whether the Ebel panel of judges set the pass mark appropriately (too high or low), or whether the cohort who sat this particular assessment could be deemed ‘non-representative’ based on experience of previous cohorts.

A possible problem with setting satisfactory boundaries which are referenced to the performance of the cohort (in this case, the median mark) is that, if the candidates in the cohort collectively agreed to ‘try less hard’ in a particular module, this might result in a lower number of fails. This possibility seems highly unlikely, particularly in a large cohort of candidates. However, the Hofstee method should counter such a possibility because the diagonal cut-off line means that the lower the effective grade boundary gets between the minimum and maximum satisfactory marks, the higher is the proportion of unsatisfactory candidates. So, applying this strategy means that candidates who might otherwise have just passed are more likely to fail.

Where competencies that can be clearly defined need to be demonstrated then a criterion-referenced form of standard setting should be advocated for determining boundaries for satisfactory performance [16, 17]. However, for assessments not concerned with competency, particularly for scientific knowledge where the difficulty and relevance are debatable, or when boundaries other than satisfactory/unsatisfactory need to be considered, then the method presented here may be more robust and has been found to be effective for cohorts ranging from 25 to 266 candidates. Indeed, as shown in Fig. 4, the MH protocol can deliver outcomes that are very similar to those generated by criterion-referenced methods, such as Ebel. Furthermore, the boundaries adopted in the MH protocol could be adjusted to correspond with local requirements.

Application of the modified Hofstee standard setting approach to resit assessments must take account of the relatively small number of candidates usually involved. In addition, a resit cohort is usually comprised of candidates with lower levels of achievement. Thus, any standard set for a resit must not be derived from student performance as the candidates involved are not likely to be representative of a complete cohort. However, there are ways in which resit assessments can have standards set for them using data from the modified Hofstee. If the resit assessment is identical to (or closely mirrors) an assessment used previously, the data from the original sitting of the assessment can be used to provide a standard using combined data, or the original standard can be retained and reapplied. Alternatively, if the resit assessment is made up of questions from a variety of previous assessments, either a hypothetical curve could be generated from the cumulative performance of these questions on previous occasions, or a generic curve for that module could be generated if there are several sets of previous data, and these show that performance is stable from year to year for that module. This would mean that new questions should not be used in a resit assessment. Clearly, if the assessment is totally new (so there are no results from previous years for the questions used in the resit assessment), an alternative method of standard setting independent of candidate performance would have to be explored.

There is clearly much scope for further analysis and development of the modified Hofstee protocol described here. For example, applying different percentage mark distances from the median for both the upper limit of the BSP and the lower limit of the BEP may be investigated for their effects on cohort performance and outcomes that may be appropriate in different circumstances. Indeed, we would recommend that this been done whenever the method is applied in a new setting in order to ensure appropriate and reliable outcomes. The modified Hofstee method could also be compared to other established standard setting methods (in addition to Ebel), such the Anghoff method.

## Conclusions

Modified Hofstee provides an objective model for calculating variable grade boundaries to take account of assessment difficulty, which, if necessary, can be converted to a fixed scale to take account of local institutional requirements. Furthermore, MH produces awards comparable to FS/CFG for the majority of candidates while treating poorer performers more fairly. It also delivers outcomes very similar to those generated by the more labour-intensive Ebel method of criterion-referenced standard setting. Modelling indicates that MH can be applied to determine an excellence/satisfactory boundary as easily as for a satisfactory/unsatisfactory boundary and this can protect against grade inflation. Furthermore, MH could be implemented as a stand-alone method of standard setting, or as part of the post-examination analysis of results for assessments for which pre-examination criterion-referenced standard setting is employed.

## Abbreviations

BEP: boundary for excellent performance
BSP: boundary for satisfactory performance
FS/CFG: formula scoring / correction for guessing
MH: modified Hofstee
PG: postgraduate
UG: undergraduate
